# 4-Chloro-5-[(5,5-dimethyl-4,5-dihydro­isoxazol-3-yl)sulfonyl­meth­yl]-3-methyl-1-(2,2,2-trifluoro­ethyl)-1*H*-pyrazole

**DOI:** 10.1107/S1600536809038471

**Published:** 2009-09-30

**Authors:** Hong-Ju Ma, Qian-Fei Zhao, Xiang-Dong Mei, Jun Ning

**Affiliations:** a Key Laboratory of Pesticide Chemistry and Application, Ministry of Agriculture, Institute of Plant Protection, Chinese Academy of Agricultural Sciences, Beijing 100193, People’s Republic of China

## Abstract

The mol­ecule of the title compound, C_12_H_15_ClF_3_N_3_O_3_S, is twisted, as indicated by the C—S—C—C torsion angle of 66.00 (18)° for the atoms linking the ring systems. An intra­molecular C—H⋯F short contact occurs. In the crystal, non-classical C—H⋯O inter­actions, one of which has a short H⋯O contact of 2.28 Å, link the mol­ecules.

## Related literature

For background to pyrazoles and their pharmacological and pharmaceutical applications, see: Hirai *et al.* (2002 ▶); Shiga *et al*. (2003 ▶); Ohno *et al.* (2004 ▶); Sabbagh *et al.* (2009 ▶); Sridhar *et al.* (2004 ▶); Zheng *et al.* (2009 ▶).
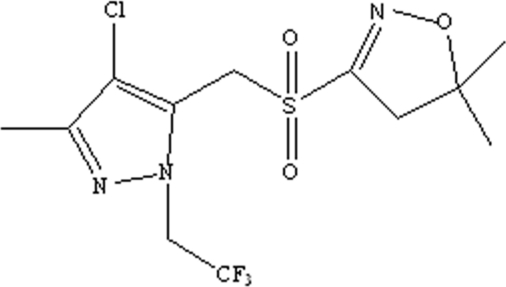


## Experimental

### 

#### Crystal data

C_12_H_15_ClF_3_N_3_O_3_S
*M* *_r_* = 373.78
Monoclinic, 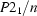

*a* = 16.034 (3) Å
*b* = 5.4319 (11) Å
*c* = 19.069 (4) Å
β = 106.71 (3)°
*V* = 1590.7 (6) Å^3^
*Z* = 4
Cu *K*α radiation
μ = 3.83 mm^−1^
*T* = 173 K
0.39 × 0.26 × 0.25 mm

#### Data collection

Rigaku R-AXIS RAPID IP diffractometer
Absorption correction: numerical (*NUMABS*; Higashi, 2003 ▶) *T*_min_ = 0.317, *T*_max_ = 0.448
11348 measured reflections
2891 independent reflections
2568 reflections with *I* > 2σ(*I*)
*R*_int_ = 0.073

#### Refinement

*R*[*F* ^2^ > 2σ(*F* ^2^)] = 0.040
*wR*(*F* ^2^) = 0.097
*S* = 1.09
2891 reflections
212 parameters
H-atom parameters constrained
Δρ_max_ = 0.39 e Å^−3^
Δρ_min_ = −0.36 e Å^−3^

### 

Data collection: *RAPID-AUTO* (Rigaku, 2001 ▶); cell refinement: *RAPID-AUTO*; data reduction: *RAPID-AUTO*; program(s) used to solve structure: *SHELXS97* (Sheldrick, 2008 ▶); program(s) used to refine structure: *SHELXL97* (Sheldrick, 2008 ▶); molecular graphics: *Mercury* (Macrae *et al.*, 2006 ▶); software used to prepare material for publication: *SHELXL97*.

## Supplementary Material

Crystal structure: contains datablocks I, global. DOI: 10.1107/S1600536809038471/hb5103sup1.cif

Structure factors: contains datablocks I. DOI: 10.1107/S1600536809038471/hb5103Isup2.hkl

Additional supplementary materials:  crystallographic information; 3D view; checkCIF report

## Figures and Tables

**Table 1:** Hydrogen-bond geometry (Å, °)

*D*—H⋯*A*	*D*—H	H⋯*A*	*D*⋯*A*	*D*—H⋯*A*
C7—H7*A*⋯F1	0.99	2.44	3.229 (3)	136
C4—H4*A*⋯O3^i^	0.98	2.60	3.325 (3)	131
C5—H5*A*⋯O2^ii^	0.99	2.31	3.146 (3)	141
C7—H7*B*⋯O1^iii^	0.99	2.28	3.265 (3)	171

Symmetry codes: (i) 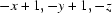; (ii) 
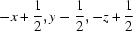
; (iii) 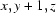.

**Fig. 1.**
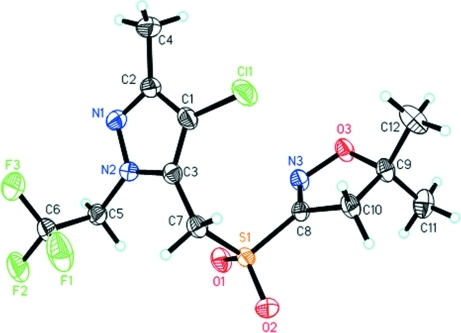
The molecular structure of the title compound, showing 50% probability displacement ellipsoids and the atom-numbering scheme.

## supplementary crystallographic information

### Comment

Pyrazoles are an important class of compounds, which possess widespread pharmacological properties in pharmaceuticals (Sridhar *et al.*, 2004; Zheng *et al.*, 2009; Sabbagh *et al.*, 2009) and agrochemicals (Shiga *et al.*, 2003; Ohno *et al.*, 2004). Various pyrazole derivatives with potent herbicidal activity have been synthesized and some are in use as herbicides such as pyrazolate, pyrazoxyfen, benzofenap, pyraflufen-ethyl, fluazolate and pyrazosulfuron-ethyl (Hirai *et al.*, 2002). Recently, the new title compound (I) was synthesized in our group with high herbicidal activity. The crystal structure of the title compound is shown in Fig. 1.

### Experimental

The title compound (0.2 g) was dissolved in acetone (50 ml) at room temperature.  Colourless blocks of (I) were obtained through slow evaporation after two weeks.

### Refinement

The H atoms were placed at calculated positions, with C—H = 0.93–0.98 Å, and refined as riding with *U*_iso_(H) = 1.2–1.5*U*_eq_(C).

### Crystal data

**Table:**

C_12_H_15_ClF_3_N_3_O_3_S	*F*(000) = 768
*M**_r_* = 373.78	*D*_x_ = 1.561 Mg m^−^^3^
Monoclinic, *P*2_1_/*n*	Cu *K*α radiation, λ = 1.54178 Å
Hall symbol: -P 2yn	Cell parameters from 11348 reflections
*a* = 16.034 (3) Å	θ = 3.2–68.2°
*b* = 5.4319 (11) Å	µ = 3.83 mm^−^^1^
*c* = 19.069 (4) Å	*T* = 173 K
β = 106.71 (3)°	Block, colourless
*V* = 1590.7 (6) Å^3^	0.39 × 0.26 × 0.25 mm
*Z* = 4	

### Data collection

**Table:**

Rigaku R-AXIS RAPID IP diffractometer	2891 independent reflections
Radiation source: rotating anode	2568 reflections with *I* > 2σ(*I*)
graphite	*R*_int_ = 0.073
ω scans	θ_max_ = 68.2°, θ_min_ = 3.2°
Absorption correction: numerical (*NUMABS*; Higashi, 2003)	*h* = −16→19
*T*_min_ = 0.317, *T*_max_ = 0.448	*k* = −6→5
11348 measured reflections	*l* = −22→20

### Refinement

**Table:**

Refinement on *F*^2^	Secondary atom site location: difference Fourier map
Least-squares matrix: full	Hydrogen site location: inferred from neighbouring sites
*R*[*F*^2^ > 2σ(*F*^2^)] = 0.040	H-atom parameters constrained
*wR*(*F*^2^) = 0.097	*w* = 1/[σ^2^(*F*_o_^2^) + (0.0203*P*)^2^ + 1.0073*P*] where *P* = (*F*_o_^2^ + 2*F*_c_^2^)/3
*S* = 1.09	(Δ/σ)_max_ < 0.001
2891 reflections	Δρ_max_ = 0.39 e Å^−^^3^
212 parameters	Δρ_min_ = −0.36 e Å^−^^3^
0 restraints	Extinction correction: *SHELXL97* (Sheldrick, 2008), Fc^*^=kFc[1+0.001xFc^2^λ^3^/sin(2θ)]^-1/4^
Primary atom site location: structure-invariant direct methods	Extinction coefficient: 0.0054 (3)

### Special details

**Table:**

Geometry. All e.s.d.'s (except the e.s.d. in the dihedral angle between two l.s. planes) are estimated using the full covariance matrix. The cell e.s.d.'s are taken into account individually in the estimation of e.s.d.'s in distances, angles and torsion angles; correlations between e.s.d.'s in cell parameters are only used when they are defined by crystal symmetry. An approximate (isotropic) treatment of cell e.s.d.'s is used for estimating e.s.d.'s involving l.s. planes.
Refinement. Refinement of *F*^2^ against ALL reflections. The weighted *R*-factor *wR* and goodness of fit *S* are based on *F*^2^, conventional *R*-factors *R* are based on *F*, with *F* set to zero for negative *F*^2^. The threshold expression of *F*^2^ > σ(*F*^2^) is used only for calculating *R*-factors(gt) *etc*. and is not relevant to the choice of reflections for refinement. *R*-factors based on *F*^2^ are statistically about twice as large as those based on *F*, and *R*- factors based on ALL data will be even larger.

### Fractional atomic coordinates and isotropic or equivalent isotropic displacement parameters (Å^2^)

**Table:**

	*x*	*y*	*z*	*U*_iso_*/*U*_eq_	
Cl1	0.44152 (4)	0.93466 (11)	0.09341 (4)	0.03950 (19)	
S1	0.36881 (3)	0.46279 (10)	0.24281 (3)	0.02353 (17)	
F1	0.10868 (10)	0.5476 (3)	0.06789 (10)	0.0610 (5)	
F2	0.05756 (9)	0.1893 (3)	0.03398 (9)	0.0584 (5)	
F3	0.09944 (9)	0.4206 (3)	−0.04032 (8)	0.0519 (5)	
O1	0.35709 (10)	0.2075 (3)	0.22345 (9)	0.0320 (4)	
O2	0.34961 (10)	0.5475 (3)	0.30760 (9)	0.0355 (4)	
O3	0.60616 (9)	0.5020 (3)	0.23839 (9)	0.0295 (4)	
N1	0.29777 (12)	0.3998 (4)	−0.01380 (10)	0.0308 (5)	
N2	0.27450 (11)	0.4064 (3)	0.04952 (10)	0.0252 (4)	
N3	0.52262 (11)	0.4070 (3)	0.22312 (10)	0.0273 (4)	
C1	0.37126 (13)	0.6956 (4)	0.06043 (13)	0.0286 (5)	
C2	0.35682 (14)	0.5758 (5)	−0.00755 (13)	0.0311 (5)	
C3	0.31789 (13)	0.5852 (4)	0.09606 (12)	0.0238 (5)	
C4	0.39748 (17)	0.6278 (6)	−0.06716 (14)	0.0478 (7)	
H4A	0.3737	0.5148	−0.1081	0.072*	
H4B	0.4606	0.6049	−0.0486	0.072*	
H4C	0.3848	0.7979	−0.0840	0.072*	
C5	0.20776 (14)	0.2398 (4)	0.05769 (12)	0.0277 (5)	
H5A	0.2192	0.1968	0.1101	0.033*	
H5B	0.2102	0.0861	0.0304	0.033*	
C6	0.11822 (15)	0.3496 (5)	0.03000 (14)	0.0358 (6)	
C7	0.30537 (13)	0.6436 (4)	0.16871 (12)	0.0252 (5)	
H7A	0.2431	0.6211	0.1654	0.030*	
H7B	0.3197	0.8193	0.1798	0.030*	
C8	0.47791 (13)	0.5464 (4)	0.25218 (11)	0.0217 (5)	
C9	0.61812 (13)	0.6994 (4)	0.29469 (12)	0.0262 (5)	
C10	0.52374 (13)	0.7681 (4)	0.29159 (13)	0.0280 (5)	
H10A	0.5161	0.7844	0.3411	0.034*	
H10B	0.5045	0.9214	0.2635	0.034*	
C11	0.66650 (17)	0.5831 (5)	0.36693 (14)	0.0415 (7)	
H11A	0.7212	0.5121	0.3632	0.062*	
H11B	0.6306	0.4531	0.3790	0.062*	
H11C	0.6791	0.7087	0.4055	0.062*	
C12	0.66893 (16)	0.9029 (5)	0.27166 (17)	0.0426 (7)	
H12A	0.7249	0.8382	0.2688	0.064*	
H12B	0.6791	1.0365	0.3077	0.064*	
H12C	0.6357	0.9658	0.2236	0.064*	

### Atomic displacement parameters (Å^2^)

**Table:**

	*U*^11^	*U*^22^	*U*^33^	*U*^12^	*U*^13^	*U*^23^
Cl1	0.0318 (3)	0.0362 (4)	0.0491 (4)	−0.0112 (2)	0.0094 (3)	0.0061 (3)
S1	0.0209 (3)	0.0256 (3)	0.0244 (3)	−0.0035 (2)	0.0071 (2)	−0.0022 (2)
F1	0.0316 (8)	0.0694 (12)	0.0759 (13)	0.0110 (8)	0.0057 (8)	−0.0260 (10)
F2	0.0300 (8)	0.0797 (13)	0.0579 (10)	−0.0223 (8)	0.0005 (7)	0.0141 (9)
F3	0.0366 (8)	0.0723 (12)	0.0408 (9)	0.0071 (8)	0.0018 (7)	0.0211 (8)
O1	0.0305 (9)	0.0225 (9)	0.0404 (10)	−0.0064 (7)	0.0060 (7)	−0.0002 (7)
O2	0.0297 (9)	0.0528 (12)	0.0271 (9)	−0.0028 (8)	0.0132 (7)	−0.0060 (8)
O3	0.0204 (8)	0.0306 (9)	0.0379 (10)	−0.0020 (6)	0.0091 (7)	−0.0091 (7)
N1	0.0277 (10)	0.0423 (12)	0.0227 (10)	0.0014 (9)	0.0077 (8)	0.0003 (9)
N2	0.0234 (9)	0.0295 (10)	0.0227 (10)	−0.0022 (8)	0.0066 (8)	0.0004 (8)
N3	0.0219 (9)	0.0281 (10)	0.0308 (10)	−0.0018 (8)	0.0060 (8)	−0.0038 (8)
C1	0.0193 (11)	0.0307 (13)	0.0338 (13)	−0.0010 (9)	0.0047 (9)	0.0068 (10)
C2	0.0226 (11)	0.0424 (14)	0.0279 (13)	0.0016 (10)	0.0068 (10)	0.0081 (10)
C3	0.0207 (10)	0.0237 (11)	0.0262 (12)	0.0025 (8)	0.0053 (9)	0.0014 (9)
C4	0.0345 (14)	0.079 (2)	0.0333 (15)	−0.0023 (14)	0.0147 (11)	0.0113 (14)
C5	0.0269 (11)	0.0280 (12)	0.0261 (12)	−0.0059 (9)	0.0043 (9)	0.0015 (9)
C6	0.0242 (12)	0.0444 (15)	0.0368 (14)	−0.0086 (11)	0.0054 (10)	0.0017 (12)
C7	0.0212 (10)	0.0238 (11)	0.0309 (12)	0.0018 (9)	0.0080 (9)	−0.0027 (9)
C8	0.0198 (10)	0.0211 (11)	0.0231 (11)	0.0010 (8)	0.0043 (9)	−0.0008 (8)
C9	0.0221 (11)	0.0208 (11)	0.0335 (13)	−0.0012 (9)	0.0048 (9)	−0.0038 (9)
C10	0.0221 (11)	0.0248 (12)	0.0365 (13)	−0.0030 (9)	0.0076 (9)	−0.0079 (10)
C11	0.0348 (14)	0.0444 (16)	0.0367 (15)	0.0013 (11)	−0.0034 (11)	−0.0013 (12)
C12	0.0295 (13)	0.0306 (14)	0.070 (2)	−0.0011 (10)	0.0186 (13)	0.0039 (13)

### Geometric parameters (Å, °)

**Table:**

Cl1—C1	1.716 (2)	C4—H4B	0.9800
S1—O2	1.4324 (16)	C4—H4C	0.9800
S1—O1	1.4333 (16)	C5—C6	1.503 (3)
S1—C8	1.766 (2)	C5—H5A	0.9900
S1—C7	1.780 (2)	C5—H5B	0.9900
F1—C6	1.329 (3)	C7—H7A	0.9900
F2—C6	1.324 (3)	C7—H7B	0.9900
F3—C6	1.344 (3)	C8—C10	1.496 (3)
O3—N3	1.386 (2)	C9—C12	1.511 (3)
O3—C9	1.490 (3)	C9—C11	1.511 (3)
N1—C2	1.327 (3)	C9—C10	1.543 (3)
N1—N2	1.363 (2)	C10—H10A	0.9900
N2—C3	1.364 (3)	C10—H10B	0.9900
N2—C5	1.444 (3)	C11—H11A	0.9800
N3—C8	1.274 (3)	C11—H11B	0.9800
C1—C3	1.374 (3)	C11—H11C	0.9800
C1—C2	1.409 (3)	C12—H12A	0.9800
C2—C4	1.491 (3)	C12—H12B	0.9800
C3—C7	1.490 (3)	C12—H12C	0.9800
C4—H4A	0.9800		
			
O2—S1—O1	119.23 (10)	F2—C6—C5	111.2 (2)
O2—S1—C8	106.40 (10)	F1—C6—C5	112.21 (19)
O1—S1—C8	109.12 (10)	F3—C6—C5	112.4 (2)
O2—S1—C7	106.96 (10)	C3—C7—S1	114.93 (15)
O1—S1—C7	109.07 (10)	C3—C7—H7A	108.5
C8—S1—C7	105.17 (10)	S1—C7—H7A	108.5
N3—O3—C9	109.60 (15)	C3—C7—H7B	108.5
C2—N1—N2	105.74 (19)	S1—C7—H7B	108.5
N1—N2—C3	112.16 (18)	H7A—C7—H7B	107.5
N1—N2—C5	118.62 (18)	N3—C8—C10	116.13 (19)
C3—N2—C5	129.16 (19)	N3—C8—S1	117.92 (16)
C8—N3—O3	108.56 (17)	C10—C8—S1	125.96 (16)
C3—C1—C2	107.0 (2)	O3—C9—C12	106.57 (19)
C3—C1—Cl1	125.86 (19)	O3—C9—C11	106.36 (18)
C2—C1—Cl1	127.13 (18)	C12—C9—C11	113.1 (2)
N1—C2—C1	109.9 (2)	O3—C9—C10	103.00 (16)
N1—C2—C4	121.7 (2)	C12—C9—C10	114.63 (19)
C1—C2—C4	128.5 (2)	C11—C9—C10	112.1 (2)
N2—C3—C1	105.2 (2)	C8—C10—C9	99.16 (17)
N2—C3—C7	125.2 (2)	C8—C10—H10A	111.9
C1—C3—C7	129.5 (2)	C9—C10—H10A	111.9
C2—C4—H4A	109.5	C8—C10—H10B	111.9
C2—C4—H4B	109.5	C9—C10—H10B	111.9
H4A—C4—H4B	109.5	H10A—C10—H10B	109.6
C2—C4—H4C	109.5	C9—C11—H11A	109.5
H4A—C4—H4C	109.5	C9—C11—H11B	109.5
H4B—C4—H4C	109.5	H11A—C11—H11B	109.5
N2—C5—C6	112.07 (19)	C9—C11—H11C	109.5
N2—C5—H5A	109.2	H11A—C11—H11C	109.5
C6—C5—H5A	109.2	H11B—C11—H11C	109.5
N2—C5—H5B	109.2	C9—C12—H12A	109.5
C6—C5—H5B	109.2	C9—C12—H12B	109.5
H5A—C5—H5B	107.9	H12A—C12—H12B	109.5
F2—C6—F1	107.5 (2)	C9—C12—H12C	109.5
F2—C6—F3	106.76 (19)	H12A—C12—H12C	109.5
F1—C6—F3	106.4 (2)	H12B—C12—H12C	109.5
			
C2—N1—N2—C3	0.1 (2)	N2—C3—C7—S1	86.0 (2)
C2—N1—N2—C5	177.64 (19)	C1—C3—C7—S1	−95.8 (3)
C9—O3—N3—C8	11.2 (2)	O2—S1—C7—C3	178.87 (16)
N2—N1—C2—C1	0.0 (2)	O1—S1—C7—C3	−50.91 (19)
N2—N1—C2—C4	−179.3 (2)	C8—S1—C7—C3	66.00 (18)
C3—C1—C2—N1	−0.2 (3)	O3—N3—C8—C10	1.4 (3)
Cl1—C1—C2—N1	180.00 (17)	O3—N3—C8—S1	−178.52 (13)
C3—C1—C2—C4	179.0 (2)	O2—S1—C8—N3	148.32 (18)
Cl1—C1—C2—C4	−0.8 (4)	O1—S1—C8—N3	18.5 (2)
N1—N2—C3—C1	−0.2 (2)	C7—S1—C8—N3	−98.41 (19)
C5—N2—C3—C1	−177.4 (2)	O2—S1—C8—C10	−31.6 (2)
N1—N2—C3—C7	178.34 (19)	O1—S1—C8—C10	−161.43 (18)
C5—N2—C3—C7	1.2 (3)	C7—S1—C8—C10	81.7 (2)
C2—C1—C3—N2	0.2 (2)	N3—O3—C9—C12	−139.32 (18)
Cl1—C1—C3—N2	−179.92 (16)	N3—O3—C9—C11	99.8 (2)
C2—C1—C3—C7	−178.3 (2)	N3—O3—C9—C10	−18.3 (2)
Cl1—C1—C3—C7	1.6 (3)	N3—C8—C10—C9	−12.4 (3)
N1—N2—C5—C6	−89.8 (2)	S1—C8—C10—C9	167.48 (16)
C3—N2—C5—C6	87.3 (3)	O3—C9—C10—C8	17.0 (2)
N2—C5—C6—F2	176.34 (19)	C12—C9—C10—C8	132.3 (2)
N2—C5—C6—F1	−63.3 (3)	C11—C9—C10—C8	−96.9 (2)
N2—C5—C6—F3	56.7 (3)		

### Hydrogen-bond geometry (Å, °)

**Table:**

*D*—H···*A*	*D*—H	H···*A*	*D*···*A*	*D*—H···*A*
C7—H7A···F1	0.99	2.44	3.229 (3)	136
C4—H4A···O3^i^	0.98	2.60	3.325 (3)	131
C5—H5A···O2^ii^	0.99	2.31	3.146 (3)	141
C7—H7B···O1^iii^	0.99	2.28	3.265 (3)	171

Symmetry codes: (i) −*x*+1, −*y*+1, −*z*; (ii) −*x*+1/2, *y*−1/2, −*z*+1/2; (iii) *x*, *y*+1, *z*.

## References

[bb1] Higashi, T. (2003). *NUMABS* Rigaku Corporation, Tokyo, Japan.

[bb2] Hirai, K., Uchida, A. & Ohno, R. (2002). *Herbicide Classes in Development*, edited by P. Boger, K. Hirai & K. Wakabyashi, pp. 179–289. Heidelberg: Springer-Verlag.

[bb3] Macrae, C. F., Edgington, P. R., McCabe, P., Pidcock, E., Shields, G. P., Taylor, R., Towler, M. & van de Streek, J. (2006). *J. Appl. Cryst.* **39**, 453–457.

[bb4] Ohno, R., Watanabe, A., Nagaoka, M., Ueda, T., Sakurai, H., Hori, M. & Hirai, K. (2004). *J* *Pestic* *Sci* **29**, 15–26.

[bb5] Rigaku (2001). *RAPID-AUTO* Rigaku Corporation, Tokyo, Japan.

[bb6] Sabbagh, O. I., Raraka, M. M., Ibrahim, S. M., Pannecouque, C., Andrei, G., Snoeck, R., Balzarini, J. & Rashad, A. A. (2009). *Eur* *J* *Med* *Chem* **44**, 3746–3753. 10.1016/j.ejmech.2009.03.038 19419804

[bb7] Sheldrick, G. M. (2008). *Acta Cryst.* A**64**, 112–122. 10.1107/S0108767307043930 18156677

[bb8] Shiga, Y., Okada, I., Ikeda, Y., Takizawa, E. & Fukuchi, T. (2003). *J* *Pestic* *Sci* **28**, 313–314.

[bb9] Sridhar, R., Perumal, P. T., Etti, S., Shanmugam, G., Ponnuswamy, M. N., Prabayathy, V. R. & Mathivanan, N. (2004). *Bioorg* *Med* *Chem* *Lett* **14**, 6035–6040. 10.1016/j.bmcl.2004.09.066 15546724

[bb10] Zheng, L. W., Wu, L. L., Zhao, B. X., Dong, W. L. & Miao, J. Y. (2009). *Bioorg* *Med* *Chem* **17**, 1957–1962. 10.1016/j.bmc.2009.01.037 19217789

